# Symptom cluster profiles and their impact on quality of life in Chinese patients with prostate cancer undergoing ADT: a cross-sectional study

**DOI:** 10.3389/fonc.2026.1867688

**Published:** 2026-07-24

**Authors:** Yingchao Xiao, Huichao Chen, Zhiqiang Chen, Juan Chen, Zhaohui Wang, Aihua Ou, Li Lan, Junwei He, Zunguang Bai, Lihong Wan

**Affiliations:** 1 Department of Urology, The Second Affiliated Hospital of Guangzhou University of Chinese Medicine, Guangzhou, China; 2 School of Nursing, Sun Yat-sen University, Guangzhou, China; 3 Nursing Department, The Second Affiliated Hospital of Guangzhou University of Chinese Medicine, Guangzhou, China; 4 Chinese Medicine Big Data Research Team, The Second Affiliated Hospital of Guangzhou University of Chinese Medicine, Guangzhou, China; 5 Urology Department, The First Affiliated Hospital of Sun Yat-sen University, Guangzhou, China

**Keywords:** androgen deprivation therapy, China, prostate cancer, quality of life, symptom clusters

## Abstract

**Background:**

Despite the growing burden of prostate cancer in China and the widespread use of androgen deprivation therapy (ADT), no validated disease-specific instrument has been used to systematically characterize symptom clusters and their impact on quality of life (QoL) in this population. This exploratory application of the newly developed Prostate Cancer Endocrine Therapy Symptom Cluster Assessment Scale (PCET-SCAS) addresses this gap.

**Objective:**

To characterize symptom cluster profiles and QoL in Chinese patients with prostate cancer undergoing ADT, examine their correlations, and identify independent predictors of QoL using a disease-specific assessment instrument.

**Methods:**

This cross-sectional study consecutively enrolled 188 patients receiving ADT at one tertiary hospital in Guangzhou, China. Symptom clusters were assessed using the PCET-SCAS, and QoL was evaluated with the EORTC QLQ-C30 and QLQ-PR25. Spearman correlation and multiple linear regression were performed for statistical analysis. This exploratory application of the PCET-SCAS aimed to characterize symptom cluster profiles and their impact on QoL in this cohort.

**Results:**

Five distinct symptom clusters were identified, including the urological-sleep, pain-digestive-nutritional, emotional-energy, endocrine therapy-related, and sexual function clusters. The sexual function cluster had the highest severity (median 2.50, IQR 2.25–3.00). Cognitive function was the lowest-scoring functional domain (median 50.00). All five clusters were significantly negatively correlated with functional domains and overall health status (r = −0.167 to −0.630, all P < 0.01), and positively correlated with symptom domains (r = 0.156 to 0.636, all P < 0.05). Univariate analysis identified age, cohabitation status, physical activity level, Karnofsky Performance Status (KPS) score, disease progression, bone metastasis, and all five symptom cluster scores as significant correlates of QoL (all P < 0.05). Multivariate regression (16 separate models, adjusted R² range: 0.139–0.541) identified symptom cluster severity as the most consistent independent predictor of QoL across domains. KPS score predicted physical, role, and social functioning but not emotional or cognitive outcomes (sexual outcomes were not analyzed in regression due to insufficient data). The emotional-energy cluster was the dominant predictor of emotional function, and the urological-sleep cluster was the strongest predictor of urinary symptoms.

**Conclusion:**

Chinese patients with prostate cancer undergoing ADT experience a multidimensional, co-occurring symptom burden across five distinct clusters, all significantly associated with impaired QoL. KPS predicted physical, role, and social functioning but not emotional or cognitive outcomes (sexual outcomes were not analyzed in regression due to insufficient data), underscoring its role as a physical performance measure rather than a global QoL indicator. These findings support the development of cluster-targeted supportive care interventions to optimize QoL in this population.

## Highlights

- What is already known on this topic: Androgen deprivation therapy (ADT) causes multi-symptom burden in prostate cancer patients, which is associated with impaired quality of life (QoL). However, no previous study has systematically applied a validated disease-specific instrument to characterize symptom clusters and their joint impact on QoL in Chinese ADT populations.
- What this study adds: Using the PCET-SCAS — a validated, China-context-specific symptom cluster assessment tool — this study identified five distinct symptom clusters in 188 Chinese patients undergoing ADT. All five clusters independently predicted QoL. The emotional-energy cluster was the strongest predictor of global health status, followed by the pain-digestive-nutritional cluster and KPS score; the endocrine therapy-related cluster was the primary predictor of hormonal treatment-related symptoms.
- What are the implications and who will benefit: Clinicians managing Chinese prostate cancer patients on ADT should integrate cluster-based symptom screening into routine care. Nursing interventions should prioritize modifiable clusters (emotional-energy, urological-sleep), and family-inclusive support strategies should be deployed given the protective role of cohabitation identified in this study.

## 1 Introduction

Globally, prostate cancer is the second most common malignancy in men, with an estimated 1.47 million new cases and 397,000 deaths in 2022 (1). In China, it has become a major public health concern: GLOBOCAN 2022 estimates indicate 134,000 new cases (ranking 6th) and 48,000 deaths (ranking 8th) among male malignancies, with age-standardized incidence and mortality rates of 9.7 per 100,000 and 3.3 per 100,000, respectively (2). The majority of Chinese patients are diagnosed at advanced or metastatic stages, with a 5-year survival rate of only 66.4% (3) — substantially lower than the 98% reported in the United States (4). Androgen deprivation therapy (ADT) is the cornerstone treatment for advanced prostate cancer, used across the disease trajectory from hormone-sensitive prostate cancer (HSPC) to castration-resistant prostate cancer (CRPC) (5–7). ADT induces a wide spectrum of treatment-related symptoms — including sexual dysfunction, hot flashes, urinary problems, and psychological distress — that substantially impair patient functioning and quality of life (QoL) (8). However, symptom burden in Chinese patients undergoing ADT may differ from Western cohorts due to unique cultural, dietary, and healthcare system factors. Notably, symptoms such as sexual dysfunction and hot flashes are often underreported in Chinese clinical settings due to cultural stigma surrounding masculinity — a pattern documented in systematic evidence showing that Asian patients experience significantly greater barriers to disclosing sexual health symptoms compared to Western counterparts (9).

The concept of a symptom cluster — defined as two or more concurrent symptoms that are related to each other and may share a common etiology (10)— has provided a theoretically grounded framework for understanding the multi-symptom experience in cancer populations. Symptom clusters are more harmful than isolated symptoms: they exert a combined, synergistic effect on patient outcomes that exceeds the impact of any individual symptom (11), and are associated with impaired functional status, reduced QoL, and worse prognosis (12–14). QoL, defined as the individual’s perception of their position in life within the context of their culture and value system (15), has become an essential outcome indicator in oncology, reflecting the cumulative impact of disease and treatment on patient well-being (16). Multiple studies have confirmed that patients with more symptom clusters and higher symptom cluster scores tend to have poorer QoL (17–19). However, existing instruments such as the EORTC QLQ-PR25 (20), were developed in Western clinical contexts and may not fully capture the culturally specific symptom presentation patterns of Chinese patients on ADT. To address this gap, our team recently developed and validated the Prostate Cancer Endocrine Therapy-Symptom Cluster Assessment Scale (PCET-SCAS), a disease-specific, multi-dimensional instrument tailored for the Chinese ADT population (21). The scale was developed through rigorous psychometric procedures—including item generation from literature review and qualitative patient interviews, expert panel evaluation, and exploratory factor analysis—to systematically capture symptoms prevalent in this population that are insufficiently covered by existing tools (e.g., hot flashes, emotional disturbances, and ADT-specific sexual dysfunction).

Although several studies have explored factors influencing QoL in prostate cancer patients undergoing ADT (17–19), none to date have comprehensively integrated demographic characteristics, clinical factors, and symptom clusters using a Chinese-context-specific instrument to examine their joint influence on QoL in this population. Accordingly, the present study applies the pre-validated five-cluster structure from the same cohort, with the following objectives, with the following objectives: (i) to characterize symptom clusters and their severity levels using the PCET-SCAS; (ii) to describe QoL across multiple functional and symptom domains; and (iii) to examine the associations between symptom clusters and QoL, and to identify key independent predictors of QoL in Chinese patients undergoing ADT.

We present this article in accordance with the STROBE reporting checklist (22).

## 2 Methods

### 2.1 Study design and setting

This was a cross-sectional descriptive study. Participants were consecutively enrolled from the urology department outpatient clinics of Guangdong Provincial Hospital of Chinese Medicine, Guangzhou, China, between January 2025 and December 2025. All assessments were conducted at a single time point at enrollment. All eligible patients presenting to the clinic during this period were invited to participate, ensuring true consecutive enrollment without selective recruitment. The study was reported in accordance with the STROBE (Strengthening the Reporting of Observational Studies in Epidemiology) guidelines.

### 2.2 Participants

Inclusion criteria were as follows: (1) histopathologically confirmed prostate cancer according to the EAU-EANM-ESTRO-ESUR-ISUP-SIOG Guidelines on Prostate Cancer (2024 version) (23); (2) undergoing androgen deprivation therapy (ADT; alone or combined with radical prostatectomy) for at least 3 months; (3) aged 18 years or older; (4) able to read and understand the questionnaire; and (5) provided informed consent and voluntarily participated.

Exclusion criteria were as follows: (1) receipt of radiotherapy or chemotherapy within the past 3 months; (2) presence of an indwelling urinary catheter; (3) history of psychiatric illness; and (4) presence of other severe comorbidities (heart failure, hematologic diseases, or other primary malignancies besides prostate cancer).

### 2.3 Sample size

The sample size was calculated using G*Power 3.1.9.7. Based on *a priori* multiple linear regression analysis with an expected medium effect size (*f²* = 0.15), significance level (*α*) = 0.05, statistical power (1−*β*) = 0.80, and 15 predictor variables (including demographic variables, clinical indicators, and the five symptom cluster scores), a minimum of 134 participants was required. Accounting for a 25% invalid response rate, the target sample size was set at 168. A total of 190 questionnaires were distributed, and 188 valid questionnaires were collected, yielding a valid response rate of 98.94%. After univariate screening, 5–9 predictor variables ultimately entered the final multivariate model, depending on the outcome variable.

### 2.4 Ethical considerations

This study was approved by the Institutional Ethics Committee of Guangdong Provincial Hospital of Chinese Medicine (Ethics No.: ZE2023-279-01). The study was conducted in accordance with the ethical principles outlined in the 2013 Declaration of Helsinki. All participants were fully informed of the study purpose, assured of data confidentiality, and provided written informed consent prior to participation.

### 2.5 Measurement instruments

#### 2.5.1 General information questionnaire

A self-designed questionnaire was used to collect demographic and clinical data, including age, place of residence, marital status, educational level, cohabitation status, payment method for medical expenses, monthly family income, height, weight, disease duration, duration of ADT, presence of comorbidities, body mass index (BMI), bone metastasis status and site, physical activity level, disease classification, treatment regimen, cancer metastasis status and site, disease progression status (from hormone-sensitive prostate cancer [HSPC] to castration-resistant disease), and Karnofsky Performance Status (KPS) score (The KPS is an 11-point scale [0–100 in 10-point increments], with 100 representing fully functional status and 0 denoting death). Based on the BMI cutoff criteria for Chinese adults issued by the National Health Commission of the People’s Republic of China, participants were categorized into four groups: underweight (BMI < 18.5 kg/m²), normal weight (18.5 ≤ BMI ≤ 23.9 kg/m²), overweight (24.0 ≤ BMI ≤ 27.9 kg/m²), and obesity (BMI ≥ 28.0 kg/m²).

#### 2.5.2 Symptom cluster assessment

Symptom clusters were assessed using the Prostate Cancer Endocrine Therapy Symptom Cluster Assessment Scale (PCET-SCAS), a disease-specific, multi-dimensional instrument developed and validated for Chinese patients undergoing ADT (21). The PCET-SCAS comprises 18 individual items distributed across five distinct symptom clusters with fixed item number ranges:

1. Urological-sleep cluster (Items 1-6): nocturia, urinary urgency, urinary incontinence, incomplete bladder emptying, difficulty falling asleep, frequent nighttime awakening;
2. Pain-digestive-nutritional cluster (Items 7-10): generalized bodily pain, poor appetite, unintended weight loss, constipation;
3. Emotional-energy cluster (Items 11-14): nervousness/tension, sadness/depression, insufficient vitality, persistent general fatigue;
4. Endocrine therapy-related symptom cluster (Items 15-16): hot flashes, excessive spontaneous perspiration;
5. Sexual function cluster (Items 17-18): reduced sexual desire, decreased frequency of morning erections

Each item is rated on a 5-point Likert scale across three dimensions (frequency, severity, distress; 0 = not at all, 1 = a little, 2 = somewhat, 3 = quite a bit, 4 = very much). If a symptom is absent, the item is scored 0 across all dimensions. If present, the item-level score is calculated as the mean of the three dimension scores (range 0–4). Cluster scores are computed as the mean of item-level scores within each cluster, and the total scale score as the mean of all item scores. Higher scores indicate more severe symptom burden. Missing items are handled by mean imputation within the cluster if ≤20% of items are missing; otherwise, the cluster score is treated as missing. The PCET-SCAS was developed and validated in the same cohort as the present study (N = 188) through exploratory and confirmatory factor analyses; the detailed item-level breakdown is provided in **Supplementary Table 1**. Therefore, the current analysis represents an exploratory application of the pre-defined five-cluster structure to examine symptom cluster-QoL associations, rather than an independent validation. The detailed item-level breakdown of all five symptom clusters, including factor loadings and Cronbach’s α values, is provided in **Supplementary Table 1**. The PCET-SCAS was developed and factor-analyzed within the present 188-patient cohort; the five-cluster structure was established through EFA and CFA in companion analyses conducted on the same sample. Therefore, the present analysis focuses on applying this validated structure to examine symptom cluster-QoL associations rather than re-evaluating the factor structure. Therefore, this study should be interpreted as an exploratory application of the instrument in a real-world clinical setting, rather than an independent validation.

#### 2.5.3 Quality of life assessment

QoL was assessed using the **EORTC QLQ-C30 (version 3.0)** and the **EORTC QLQ-PR25**.

The EORTC QLQ-C30 (version 3.0) (24) and the EORTC QLQ-PR25 (20), both previously validated in Chinese populations (25, 26) were used to assess health-related QoL.

The QLQ-C30 consists of five functional scales (physical, role, cognitive, emotional, and social functioning), three symptom scales (fatigue, pain, and nausea/vomiting), overall health status, and six single-item measures (dyspnea, insomnia, appetite loss, constipation, diarrhea, and financial difficulties). The QLQ-PR25 includes three symptom domains (urinary, bowel, and treatment-related symptoms) and one sexual functioning domain. Raw scores were linearly transformed to 0–100 standardized scores. For functional scales and overall health status, higher scores indicate better functioning; for symptom scales, higher scores indicate more severe symptoms. The Chinese version of QLQ-C30 has demonstrated acceptable psychometric properties (Cronbach’s α= 0.875) (25).

### 2.6 Data collection

All investigators were uniformly trained before data collection. Questionnaires were administered through face-to-face interviews conducted in the urology outpatient clinic. Before the survey, the purpose and significance of the study were explained to patients or their families, and written informed consent was obtained. Patients who were unable to complete the questionnaire independently were assisted by a trained investigator using a standardized verbal explanation protocol. All completed questionnaires were verified on-site immediately upon completion to ensure completeness and accuracy.

### 2.7 Statistical analysis

Statistical analysis was performed using SPSS 26.0 (IBM, USA). Normality of continuous variables was assessed using Kolmogorov-Smirnov tests, supplemented by P-P plots and Q-Q plots; given the non-normal distribution of QoL scores, continuous data were expressed as medians with interquartile ranges (P50 [P25, P75]). Categorical data were presented as frequencies and proportions. The Wilcoxon rank-sum test (Mann-Whitney U test) was used for two-group comparisons, and the Kruskal-Wallis H test was used for multi-group comparisons of QoL scores across patient characteristics. Spearman correlation analysis was used The magnitude of Spearman correlation coefficients was interpreted according to established guidelines as follows: negligible or very low correlation (r_s_ < 0.30), low correlation (0.30 ≤ r_s_ < 0.50), moderate correlation (0.50 ≤ r_s_ < 0.70), high correlation (0.70 ≤ r_s_ < 0.90), and very high correlation (r_s_ ≥ 0.90) (27). Model fit was evaluated using the coefficient of determination (R²) and adjusted R². Overall model significance was assessed using the F-test.

Multiple linear regression analysis (stepwise forward selection) was performed to identify independent predictors of QoL. Predictors entered into multivariate modeling included age (retained *a priori*), physical activity level, KPS score, disease progression status, cohabitation status, and the five symptom cluster scores (Urological-sleep, Pain-digestive-nutritional, emotional-energy, Endocrine therapy-related, Sexual function). Variables with *P* < 0.05 in univariate analysis were retained for multivariate modeling. Multivariate linear regression models were constructed for each QoL domain as the dependent variable (16 models in total), with the QLQ-C30 Global Health Status (GHS) score and QLQ-PR25 hormonal treatment-related symptoms serving as the primary and secondary outcomes of interest, respectively. A secondary model was constructed for QLQ-PR25 hormonal treatment-related symptoms, given its direct clinical relevance to the ADT context. Multicollinearity among independent variables was assessed using the Variance Inflation Factor (VIF), with VIF < 5 indicating acceptable levels (no significant multicollinearity was identified; all VIF values ranged from 1.02 to 3.47). All statistical tests were two-sided, with *P* < 0.05 considered statistically significant. Given the exploratory nature of this study, which aimed to characterize symptom cluster-QoL associations across multiple clinically relevant domains, no formal correction for multiple comparisons was applied. Findings should be interpreted cautiously as exploratory, and the full pattern of associations is reported transparently to inform future confirmatory research (28, 29). Residual diagnostics confirmed approximate normality for all models upon visual inspection of Q-Q plots, and no extreme outliers exerted undue leverage.

Missing data for PSA were handled using mean imputation; all other variables were analyzed using complete case analysis. Sensitivity analyses comparing participants with and without missing PSA values confirmed no significant systematic differences in baseline characteristics (all *P* > 0.05).

## 3 Results

### 3.1 Patient characteristics

A total of 188 patients were included in the final analysis. As shown in **Table 1**, the majority of patients were elderly (aged ≥60 years: 93.62%), with the highest proportion aged 70–79 years (42.55%). The mean age was 72.76 ± 9.03 years (range: 47–93 years). Most patients resided in urban areas (88.30%), were married (92.55%), and lived with their spouse or children (91.49%). Educational level was predominantly junior high school (33.51%) and senior high school (30.32%). The majority (82.45%) had medical insurance coverage. Regarding clinical characteristics, the majority had metastatic disease (mHSPC: n=131, 69.68%; mCRPC: n=43, 22.87%; non-metastatic HSPC: n=14, 7.45%); bone metastasis was present in 67.55% (n=127) of patients. Serum prostate-specific antigen (PSA) at initial diagnosis was >10 ng/mL in 68.65% of patients; current PSA ≤4 ng/mL in 68.11% of patients, indicating adequate biochemical control. The median ADT duration was 24.0 months (IQR: 12.0-60.0) for the overall cohort. By disease subset, the median ADT duration was 23.0 months (IQR: 8.0-48.0) for non-metastatic HSPC, 36.0 months (IQR: 10.0–47.0) for mHSPC, and 47.0 months (IQR: 4.0-62.0) for mCRPC. The most common treatment modality was ADT alone (60.64%), and 48.94% had comorbid hypertension. Most patients had good functional status (79.30% had KPS 80–100). Subgroup analyses confirmed that prior RP did not significantly affect QoL outcomes.

**Table 1:** Baseline characteristics of patients undergoing androgen deprivation therapy.

Variable	n (%)	Variable	n (%)	Variable	n (%)
Age (years)		Educational level		Initial PSA* (ng/mL)	
47–59	13 (6.91)	Primary school or below	28 (14.89)	0.04–4.00	9 (4.86)
60–69	50 (26.60)	Junior high school	63 (33.51)	4.01–10.00	14 (7.57)
70–79	80 (42.55)	Senior high/vocational	57 (30.32)	>10.00	127 (68.65)
80–93	45 (23.94)	College/university	40 (21.28)	Missing	35 (18.92)
Residence		Payment method		Current PSA* (ng/mL)	
Urban	166 (88.30)	Public medical care	21 (11.17)	≤4.00	126 (68.11)
Rural	22 (11.70)	Medical insurance	155 (82.45)	4.01–10.00	9 (4.86)
Marital status		Self-payment	12 (6.38)	>10.00	22 (11.89)
Married	174 (92.55)	Family income (yuan/month)		Missing	28 (15.14)
Unmarried/divorced/widowed	14 (7.45)	≤2999	21 (11.17)	Disease classification**	
Living arrangement		3000–4999	50 (26.60)	Non-metastatic HSPC	14 (7.45)
With spouse/children	172 (91.49)	5000–7999	84 (44.68)	Metastatic HSPC	131 (69.68)
Living alone	16 (8.51)	≥8000	33 (17.55)	Metastatic CRPC	43 (22.87)
Disease duration (months)		BMI (kg/m²)			
3–12	53 (28.19)	18.50–23.99	112 (59.57)	Treatment regimen	
13–24	27 (14.37)	24.00–27.99	56 (29.79)	ADT alone	114 (60.64)
25–36	23 (12.23)	28.00–36.89	13 (6.91)	Surgery + ADT	74 (39.36)
37–48	18 (9.57)	ADT duration (months)			
49–60	20 (10.64)	3–12	44 (23.40)	Bone metastasis	
61–205	47 (25.00)	13–24	32 (17.02)	No	61 (32.45)
Hypertension		25–36	18 (9.57)	Yes	127 (67.55)
No	96 (51.06)	37–48	19 (10.12)	KPS score	
Yes	92 (48.94)	49–60	27 (14.36)	30–70	39 (20.70)
Comorbid diabetes		61–152	48 (25.53)	80–100	149 (79.30)
No	154 (81.91)	Physical activity***			
Yes	34 (18.09)	Inactive	66 (35.11)	Disease progression	
Comorbid CHD		Insufficiently active	96 (51.06)	No	127 (67.55)
No	160 (85.11)	Active	26 (13.83)	Yes	61 (32.45)
Yes	28 (14.89)				

(ADT) for Prostate Cancer (n = 188).

* PSA, prostate-specific antigen (normal adult male reference: <4 ng/mL). **Disease classification based on EAU-EANM-ESTRO-ESUR-ISUP-SIOG Guidelines on Prostate Cancer (2024 version). *** Physical activity was classified according to the International Physical Activity Questionnaire (IPAQ) guidelines (≤75 min/week vigorous activity), insufficiently active, and active. ADT, androgen deprivation therapy; BMI, body mass index; CHD, coronary heart disease; CRPC, castration-resistant prostate cancer; ADT, Androgen Deprivation Therapy; HSPC, hormone-sensitive prostate cancer; KPS, Karnofsky Performance Status; PSA, prostate-specific antigen.

The median follow-up time from ADT initiation to data collection was 18.5 months (IQR: 9.2–32.1 months) for the overall cohort. The RP + ADT subgroup had a median follow-up of 16.2 months (IQR: 8.5–28.4 months), and the ADT-only subgroup had a median follow-up of 20.1 months (IQR: 10.3–34.7 months). A Kruskal-Wallis test confirmed no significant difference in follow-up duration between subgroups (P = 0.42).

### 3.2 Symptom cluster scores

Symptom cluster scores are presented in **Table 2**. Symptom cluster severity scores ranked from highest to lowest as follows: sexual function cluster (median 2.50, IQR 2.25–3.00), emotional-energy cluster (median 1.17, IQR 0.25–1.88), endocrine therapy-related cluster (median 1.00, IQR 0.00–2.00), urological-sleep cluster (median 0.94, IQR 0.39–1.98), and pain-digestive-nutritional cluster (median 0.25, IQR 0.00–0.71). Notably, within the sexual function cluster, both items—reduced sexual desire and decreased morning erections—had a prevalence of 100%. The median severity score (3.00, IQR 3.00–4.00) was substantially higher than the median distress score (1.50, IQR 1.00–3.00), indicating that while patients experienced severe sexual dysfunction attributable to ADT, the self-reported subjective distress was comparatively moderate. This pattern likely reflects both the profound impact of androgen deprivation on sexual function and a degree of response shift. Furthermore, the lack of significant correlation between the sexual function cluster and sexual quality of life (QoL) may reflect high between-patient variance in this domain (given the 100% prevalence of dysfunction) rather than a true absence of cluster effects on sexual well-being. Within the urological-sleep cluster, items showed heterogeneous prevalence rates (range: 33%–70%), with low-frequency items contributing minimal within-cluster variance. Consequently, the median severity and distress scores converged at 0.83, a pattern attributable to right-skewed item distributions rather than any data quality issue.

**Table 2:** Symptom cluster scores in patients undergoing androgen deprivation therapy (ADT) for prostate cancer (n = 188).

Symptom cluster	Items (n)	Dimension Total score M [P25, P75]	Mean score M [P25, P75]	Severity score M [P25, P75]	Distress score M [P25, P75]
Urological-Sleep	6	5.66 [2.33, 11.83]	0.94 [0.39, 1.98]	0.83 [0.33, 1.83]	0.83 [0.33, 1.83]*
Pain-Digestive-Nutritional	4	1.00 [0.00, 2.84]	0.25 [0.00, 0.71]	0.25 [0.00, 0.63]	0.25 [0.00, 0.50]
Emotional-Energy	4	4.66 [1.00, 7.50]	1.17 [0.25, 1.88]	1.00 [0.25, 1.75]	1.00 [0.25, 1.50]
Endocrine Therapy-Related	2	2.00 [0.00, 4.00]	1.00 [0.00, 2.00]	1.00 [0.00, 2.00]	0.50 [0.00, 2.00]
Sexual Function	2	5.00 [4.50, 6.00]	2.50 [2.25, 3.00]	3.00 [3.00, 4.00]	1.50 [1.00, 3.00]

Scores range from 0 to 4; higher scores indicate more severe symptom burden. M, median; P25, 25th percentile; P75, 75th percentile. *Within the Urological-Sleep cluster, lower-prevalence items (e.g., incontinence, incomplete emptying) contributed minimal within-cluster variance, resulting in right-skewed distributions and convergent median severity and distress scores at the cluster level. This reflects genuine distributional properties of the cluster rather than data entry error.

Note: Higher scores indicate more severe symptom burden. Scoring: frequency (1–4), severity (1–4), and distress (0–4) are recoded to 0–4 scales, then averaged within each cluster.

### 3.3 Quality of life scores

QoL scores are presented in **Table 3**. In the EORTC QLQ-C30, among the five functional scales, cognitive function had the lowest score (median 50.00, IQR 50.00–83.33), while among the three symptom scales, fatigue had the highest score (median 33.33, IQR 11.11–44.44). Among the six single-item symptoms, insomnia and financial difficulties had the highest scores (both median 33.33, IQR 0.00–33.33). In the QLQ-PR25, the highest score was observed in the treatment-related symptoms domain (median 33.33, IQR 0.00–33.33). Within the sexual functioning domain, sexual interest and pleasure had the highest scores (both median 100.00, IQR 100.00–100.00) while sexual QoL had the lowest score (median 0.00, IQR 0.00–0.00). Only 8 patients (4.26%) reported sexual activity within the past 4 weeks and completed the sexual functioning sections. This is likely attributable to multiple factors: (1) cultural stigma surrounding sexual health discussions among elderly Chinese men, leading to systematic underreporting; (2) the advanced mean age of our cohort (72.76 ± 9.03 years), with most patients having ceased sexual activity independent of their cancer diagnosis; and (3) the direct pharmacological effect of ADT, which suppresses sexual desire and erectile function. Accordingly, sexual functioning data are reported descriptively only and were excluded from inferential analyses. The sexual functioning domain is annotated accordingly in **Table 3**.

**Table 3:** Quality of life scores (QLQ-C30 and QLQ-PR25) in patients undergoing androgen deprivation therapy (ADT) for prostate cancer (n = 188).

QLQ-C30 domain	Score M [P25, P75]	QLQ-PR25 domain	Score M [P25, P75]
Overall health status	58.33 [50.00, 66.67]	Urinary symptoms	22.22 [11.11, 33.33]
Functional domains		Bowel symptoms	0.00 [0.00, 8.33]
Physical function	73.33 [60.00, 86.67]	Treatment-related symptoms*	33.33 [0.00, 33.33]
Role function	83.33 [66.67, 100.00]	Sexual interest	100.00 [100.00, 100.00]
Emotional function	83.33 [75.00, 100.00]	Sexual pleasure	100.00 [100.00, 100.00]
Cognitive function	50.00 [50.00, 83.33]	Sexual QoL	0.00 [0.00, 0.00]
Social function	66.67 [54.17, 79.17]		
Symptom domains			
Fatigue	33.33 [11.11, 44.44]		
Nausea and vomiting	0.00 [0.00, 0.00]		
Pain	0.00 [0.00, 16.67]		
Single items			
Dyspnea	0.00 [0.00, 33.33]		
Insomnia	33.33 [0.00, 33.33]		
Appetite loss	0.00 [0.00, 0.00]		
Constipation	0.00 [0.00, 33.33]		
Diarrhea	0.00 [0.00, 0.00]		
Financial difficulties	33.33 [0.00, 33.33]		

*n = 8/188 (4.26%); only 8 patients who reported sexual activity within the past 4 weeks completed this domain. For QLQ-C30 functional domains and overall health status: higher scores = better functioning. For QLQ-C30 symptom domains and QLQ-PR25 symptom domains: higher scores = more severe symptoms.

### 3.4 Correlation between symptom clusters and QoL

The results of Spearman correlation analysis between symptom clusters and QoL are presented in **Table 4** and **5**. All five symptom clusters were significantly negatively correlated with functional domains and overall health status on the QLQ-C30 (*r* = −0.630 to −0.167, *P* < 0.01), and significantly positively correlated with symptom domains (*r* = 0.156 to 0.636, *P* < 0.01). The pain-digestive-nutritional symptom cluster had the broadest association, correlating significantly with all 15 QoL domains. The emotional-energy cluster showed the strongest negative correlation with emotional function (*r* = −0.630, *P* < 0.01). The sexual function symptom cluster had the narrowest association (6/15 domains). In the QLQ-PR25, the urological-sleep and pain-digestive-nutritional clusters were significantly positively correlated with urinary, bowel, and treatment-related symptoms (*P* < 0.01). The emotional-energy cluster (*r* = 0.501) and endocrine therapy-related cluster (*r* = 0.608) showed strong positive correlations with treatment-related symptoms. No significant correlation was found between any symptom cluster and sexual interest (*P* > 0.05).

**Table 4:** Correlation between symptom clusters and QLQ-C30 domains (Spearman *r*, n = 188).

Symptom cluster	Overall health *(r)*	Physical function *(r)*	Role function *(r)*	Emotional function *(r)*	Cognitive function *(r)*	Social function *(r)*	Fatigue *(r)*	Nausea and vomiting. *(r)*	Pain *(r)*	Dyspnea *(r)*	Insomnia *(r)*	Appetite loss *(r)*	Constipation *(r)*
Urological-Sleep	-0.406**	-0.457**	-0.507**	-0.282**	-0.369**	-0.383**	0.488**	0.168*	0.172*	0.465**	0.636**	0.212**	0.180*
Pain-Digestive-Nutritional	-0.507**	-0.596**	-0.568**	-0.320**	-0.349**	-0.497**	0.636**	0.334**	0.543**	0.401**	0.386**	0.577**	0.501**
Emotional-Energy	-0.579**	-0.507**	-0.481**	-0.630**	-0.410**	-0.391**	0.610**	0.176*	0.229**	0.237**	0.239**	0.204**	0.147*
Endocrine Therapy-Related	-0.267**	-0.182*	-0.245**	-0.272**	-0.102	-0.198**	0.257**	0.121	0.211**	0.089	0.156*	0.051	0.049
Sexual Function	-0.254**	-0.109	-0.037	-0.167*	-0.196**	-0.071	0.098	0.116	0.069	0.001	0.139	0.218**	0.171*

** P < 0.01; * P < 0.05. Functional domain correlations are negative (higher symptom scores → lower function); symptom domain correlations are positive (higher symptom scores → more severe symptoms). n = 188 for all analyses.

**Table 5:** Correlation between symptom clusters and QLQ-PR25 domains (Spearman *r*, n = 188).

Symptom cluster	Urinary symptoms (r)	Bowel symptoms (r)	Treatment-related symptoms (r)	Sexual interest (r)
Urological-Sleep	0.783**	0.275**	0.422**	0.050
Pain-Digestive-Nutritional	0.439**	0.313**	0.488**	0.044
Emotional-Energy	0.348**	0.141	0.501**	0.044
Endocrine Therapy-Related	0.055	0.096	0.608**	0.092
Sexual Function	0.022	0.163*	0.165*	0.026

** P < 0.01; * P < 0.05. n = 188; sexual functioning domain (sexual pleasure, sexual QoL) not analyzed due to insufficient data (n = 8/188, 4.26%).

This negligible correlation between the sexual function cluster and the sexual interest item (*r* = 0.026) reflects the conceptual distinction between objective functional impairment (as captured by the PCET-SCAS sexual function cluster, which assesses erectile dysfunction and loss of libido as symptom burden) and subjective sexual desire (as captured by the EORTC QLQ-PR25 single item, which assesses interest in sexual activity). These two constructs are complementary but not interchangeable; a patient may retain sexual interest despite experiencing erectile dysfunction, or conversely, may lose interest due to psychological or relational factors independent of functional capacity. Low correlations between sexual function and sexual interest have been previously documented in prostate cancer populations (30, 31). Accordingly, this finding should not be interpreted as evidence of poor construct validity of the PCET-SCAS, but rather as a reflection of the multidimensional nature of sexual health.

### 3.5 Factors influencing QoL - univariate analysis

Univariate analysis (Kruskal-Wallis H test and Mann-Whitney U test) revealed significant differences in Global Health Status (GHS) scores according to the following variables (all *P* < 0.05): age, cohabitation status, physical activity level, KPS score, disease progression status (HSPC vs. CRPC), bone metastasis, and coronary heart disease. All five symptom cluster scores were significantly associated with QoL across multiple domains (all *P* < 0.01). Additionally, urban/rural residence showed significant associations with cognitive, emotional, and social function (*P* < 0.05), though this finding should be interpreted cautiously given the unequal group sizes (urban: *n* = 166; rural: *n* = 22). Variables significant in univariate analysis, together with age (retained *a priori*), were entered into multivariate regression.

CHD was a significant univariate predictor of physical functioning (P < 0.05) but was not retained in the final multivariate model after controlling for other covariates, suggesting that its effect is mediated by physical symptom clusters or other comorbidities.

### 3.6 Factors influencing QoL - multivariate regression analysis

Multiple linear regression analysis was performed to identify independent predictors of QoL. Separate multivariate linear regression models were constructed for each QoL domain as the dependent variable (16 models in total). Independent variables entered into all models included age and all variables that showed statistical significant in univariate analysis (*P* < 0.05). The regression results are summarized in **Table 6**. Variance inflation factor (VIF) values ranged from 1.02 to 3.47 across all models, suggesting no severe multicollinearity between predictors. Given that multiple regression models were established without formal correction for multiple comparisons, all derived associations should be interpreted cautiously to avoid overinterpreting inflated Type I error risk.

**Table 6:** Multivariate linear regression analysis of factors influencing quality of life (n=188).

Dependent variable (QoL)		Independent variable	*β*	95% CI	P-value	R²/Adj. R²	F
QLQ-C30	Overall health status	KPS score	0.185	[0.077, 0.293]	<0.001	0.523/0.510	39.989
		Disease progression (yes)	-0.140	[-0.227, -0.053]	0.011		
		Emotional-energy cluster	-0.389	[-0.457, -0.038]	<0.001		
		Pain-digestive-nutritional cluster	-0.200	[-0.323, -0.052]	0.004		
		Urological-sleep cluster	-0.139	[-0.268, -0.055]	0.027		
	Physical function	KPS score	0.387	[0.272, 0.502]	<0.001	0.481/0.473	56.942
		Pain-digestive-nutritional cluster	-0.325	[-0.578, -0.268]	<0.001		
		Emotional-energy cluster	-0.207	[-0.382, -0.108]	0.001		
	Role function	KPS score	0.236	[0.102, 0.370]	0.001	0.518/0.507	49.167
		Physical activity (active)	0.160	[0.046, 0.274]	0.020		
		Urological-sleep cluster	-0.141	[-0.424, -0.046]	0.020		
		Pain-digestive-nutritional cluster	-0.423	[-0.524, -0.076]	<0.001		
	Emotional function	Emotional-energy cluster	-0.588	[-0.703, -0.473]	<0.001	0.411/0.404	64.431
		Endocrine therapy-related cluster	-0.125	[-0.283, -0.062]	0.038		
	Cognitive function	Cohabitation (with spouse/children)	0.151	[0.039, 0.263]	0.016	0.288/0.272	18.478
		Emotional-energy cluster	-0.260	[-0.344, -0.049]	<0.001		
		Sexual function cluster	-0.174	[-0.278, -0.070]	0.008		
		Urological-sleep cluster	-0.285	[-0.428, -0.036]	<0.001		
	Social function	KPS score	0.217	[0.094, 0.340]	<0.001	0.367/0.357	35.621
		Pain-digestive-nutritional cluster	-0.392	[-0.517, -0.140]	<0.001		
		Emotional-energy cluster	-0.172	[-0.289, -0.056]	0.013		
	Fatigue	Pain-digestive-nutritional cluster	0.420	[0.261, 0.579]	<0.001	0.546/0.536	55.108
		Urological-sleep cluster	0.287	[0.111, 0.462]	0.002		
		KPS score	0.175	[0.057, 0.292]	0.004		
	Nausea and Vomiting	Pain-digestive-nutritional cluster	0.323	[0.077, 0.460]	<0.001	0.169/0.160	18.753
		KPS score	-0.177	[-0.317, -0.037]	0.013		
	Pain	Pain-digestive-nutritional cluster	0.632	[0.254, 0.760]	<0.001	0.319/0.311	43.286
		Urological-sleep cluster	-0.161	[-0.318, -0.004]	0.025		
	Dyspnea	Urological-sleep cluster	0.176	[0.023, 0.329]	0.019	0.255/0.243	21.036
		KPS score	-0.265	[-0.412, -0.043]	<0.001		
		Pain-digestive-nutritional cluster	0.233	[0.063, 0.367]	0.003		
	Insomnia	Urological-sleep cluster	0.447	[0.429, 0.737]	<0.001	0.314/0.307	42.331
		Pain-digestive-nutritional cluster	0.177	[0.129, 0.451]	0.014		
	Appetite loss	Pain-digestive-nutritional cluster	0.714	[0.279, 1.149]	<0.001	0.537/0.526	52.97
		KPS score	-0.217	[-0.334, -0.057]	<0.001		
		Urological-sleep cluster	0.241	[0.017,0.491]	<0.001		
		Sexual function cluster	-0.194	[-0.311, -0.091]	<0.001		
	Constipation	Pain-digestive-nutritional cluster	0.482	[0.217, 0.625]	<0.001	0.260/0.252	32.457
		Sexual function cluster	-0.151	[-0.417, -0.092]	0.018		
QLQ-PR25	Urinary symptoms	Urological-sleep cluster	0.587	[0.479, 0.695]	<0.001	0.446/0.440	74.352
		KPS score	-0.215	[-0.311, -0.119]	<0.001		
	Bowel symptoms	Pain-digestive-nutritional cluster	0.311	[0.173, 0.450]	<0.001	0.148/0.139	16.058
		Sexual function cluster	-0.217	[-0.368, -0.065]	0.002		
	Hormonal treatment	Endocrine therapy-related cluster	0.465	[0.361, 0.568]	<0.001	0.554/0.541	45.14
		Pain-digestive-nutritional cluster	0.205	[0.079, 0.332]	0.001		
		Emotional-energy cluster	0.133	[0.011, 0.255]	0.033		
		Urological-sleep cluster	0.152	[0.034, 0.269]	0.012		
		Sexual function cluster	0.132	[0.030, 0.234]	0.012		

Note: Data are presented as unstandardized β coefficients with 95% confidence intervals. Separate multivariate linear regression models were constructed for each QoL domain as the dependent variable (16 models in total). Each row represents a significant independent predictor (P* < 0.05). All models were statistically significant at P < 0.001. Variance inflation factor (VIF) values ranged from 1.02 to 3.47, indicating acceptable multicollinearity. Durbin-Watson statistics (1.382–2.065) indicated no significant autocorrelation of residuals.

Abbreviations: KPS, Karnofsky Performance Status; CI, confidence interval; β, unstandardized regression coefficient; R², coefficient of determination; Adj. R², adjusted R².

### 3.7 Reliability of the assessment instruments

The reliability of the assessment instruments was evaluated in the present cohort. The PCET-SCAS demonstrated excellent internal consistency, with a Cronbach’s α of 0.950 for the total scale and subscale α values ranging from 0.813 (endocrine therapy-related cluster) to 0.918 (urological-sleep cluster). Test-retest reliability over a two-week interval was satisfactory, with an intraclass correlation coefficient (ICC) of 0.717 (95% CI: 0.642–0.801). For the EORTC QLQ-C30 and QLQ-PR25, Cronbach’s α coefficients were 0.881 and 0.828, respectively.

To assess the robustness of our findings, we conducted several sensitivity analyses. First, we re-analyzed all multivariate models using robust regression with Huber-White sandwich estimators and quantile regression (median, 25th and 75th percentiles). The pattern of significant predictors and the direction of effects remained consistent with the OLS results. Second, we applied the Benjamini-Hochberg false discovery rate (FDR) correction to the multivariate model P-values. After FDR adjustment (q < 0.05), the pain-digestive-nutritional cluster remained significantly associated with 9 of 12 primary QoL outcomes, and the emotional distress cluster remained significant for 8 of 12 outcomes. The gastrointestinal and urinary-sleep clusters remained significant for 6 and 4 outcomes, respectively. Third, a sensitivity analysis including only patients with complete PSA data (n=168) yielded results qualitatively identical to the main analysis using mean imputation. These sensitivity analyses support the robustness of our primary findings.

## 4 Discussion

This study provides a comprehensive characterization of symptom cluster profiles and their impact on quality of life (QoL) in Chinese patients with prostate cancer undergoing androgen deprivation therapy (ADT), a population that has been substantially underrepresented in the international symptom cluster literature. Through a cross-sectional survey of 188 patients recruited consecutively from the urology outpatient clinics of Guangdong Provincial Hospital of Chinese Medicine, Guangzhou, China, between January and December 2025, we identified five distinct symptom clusters, documented their pervasive cross-domain associations with QoL, and established the independent predictors of QoL outcomes through multivariate regression analysis. The following discussion contextualizes these findings within the existing literature, addresses their clinical implications, and acknowledges the limitations of the present analysis.

### 4.1 Symptom cluster profiles and cultural context

The heterogeneity of ADT agents used in clinical practice—particularly the use of next-generation anti-androgens (e.g., enzalutamide, abiraterone) in CRPC patients—may have introduced variability in symptom profiles. Next-generation anti-androgens have distinct toxicity profiles (e.g., hypertension and decreased cognitive function with enzalutamide) that could directly affect patients’ quality of life. In the present study, androgen deprivation therapy (ADT) administered to all enrolled patients was predominantly based on luteinizing hormone-releasing hormone (LHRH) agonists. A national survey among Chinese urologists has validated that LHRH agonists serve as the mainstream regimen for ADT in domestic clinical practice, with predominant clinical preference for long-acting formulations (32). Notably, LHRH antagonists were not commercially available or accessible at our institution throughout the entire data collection period. Given this objective clinical condition, the ADT exposure in our study cohort exhibited favorable homogeneity in terms of drug category, which effectively minimized the potential confounding bias derived from heterogeneous ADT regimens and guaranteed the reliability of subsequent analytical results.

The pain-digestive-nutritional symptom cluster had the lowest severity (median 0.25), suggesting that gastrointestinal symptoms and pain are relatively less prevalent in this specific treatment population compared to other cancer groups undergoing more aggressive treatments. The emotional-energy symptom cluster (median 1.17) reflects the psychological burden of cancer diagnosis and treatment, including anxiety, depression, and fatigue, which are common among cancer patients on endocrine therapy (18, 19). The endocrine therapy-related symptom cluster (median 1.00) captures treatment-specific symptoms such as hot flashes and sweating, which are direct consequences of androgen suppression (33). The urological-sleep symptom cluster (median 0.94) likely reflects the combined effects of urinary symptoms related to the disease itself and sleep disturbances secondary to hot flashes and nocturia, consistent with the bidirectional relationship between lower urinary tract symptoms and sleep quality reported in Chinese male populations (34).

From a Chinese cultural perspective, several findings warrant particular attention. First, sexual symptoms—particularly sexual dysfunction—may be underreported in Chinese clinical settings due to cultural norms surrounding masculinity, a pattern that is discussed in greater detail in the following section. Second, the consistent protective effect of cohabitation on QoL aligns with traditional Chinese family-centered care values, where spousal and family support form the backbone of long-term illness management. This cultural strength should be actively leveraged through family-inclusive nursing interventions and peer support programs tailored to the Chinese context. Third, the predominance of traditional Chinese dietary practices among patients may interact with ADT-related gastrointestinal symptoms in ways that differ from Western populations, warranting further interdisciplinary investigation.

The five-cluster structure of the PCET-SCAS was established during the instrument development phase through exploratory and confirmatory factor analyses (21). In the present study, we applied this pre-defined cluster structure to characterize symptom burden in our cohort. Given that the PCET-SCAS development and validation have been reported in a companion publication, the present analysis focuses on applying this validated framework to explore the clinical significance of symptom clusters and their relationship with QoL, rather than re-evaluating the factor structure.

### 4.2 Physiological trajectory of sexual function on ADT

The sexual function symptom cluster had the highest severity score (median 2.50/4.00), with both included symptoms — reduced sexual desire and decreased morning erections — reaching a prevalence of 100%. This finding is consistent with the well-established effects of ADT, which causes a 70–90% reduction in testosterone levels within 1–3 months of treatment initiation, directly and predictably impairing sexual function (35). The discrepancy between the high severity score (3.00) and the relatively lower distress score (1.50) in this cluster may reflect multiple mechanisms operating simultaneously. Beyond patients’ psychological adaptation to sexual dysfunction as an expected treatment side effect, two methodological factors likely contribute to this pattern. First, the 100% prevalence rate effectively reduces between-patient variance, which may compress the magnitude of correlations between the sexual function cluster and other QoL domains — a form of restricted range effect whereby the absence of variability in the predictor limits the detection of true associations. Second, in the Chinese cultural context — particularly given the advanced median age of our cohort (72 years) and the sensitive nature of sexual health topics — patients may be more likely to underreport distress associated with sexual dysfunction, even in face-to-face questionnaire administration settings (9).

Physiological Time Trajectory of Sexual Function on ADT: A Phased Model.

ADT-induced sexual dysfunction follows a distinct, phased time course rather than a linear progression with treatment duration. Understanding this trajectory is essential for interpreting the lack of correlation between ADT duration and sexual function symptoms observed in our study. Phase 1 (1–3 months): Testosterone decreases by 70–90% to castrate level (<50 ng/dL) within 1–3 months of ADT initiation, leading to rapid decline in libido and erectile function during this window. Phase 2 (3–12 months): Continuous structural organ damage accumulates — corpus cavernosum smooth muscle apoptosis, venous leak, and vascular endothelial dysfunction progress. By 12 months, objective sexual function (libido, erectile function) reaches its nadir (floor effect). Longitudinal cohort studies have documented that erectile hardness significantly declines by 3 months, the proportion of men able to complete intercourse significantly decreases by 6 months, and structural corporal damage peaks at 12 months. Phase 3 (≥12 months): Further structural damage plateaus because the majority of apoptosis-sensitive smooth muscle has already been depleted. Even with continued castrate-level testosterone, objective sexual function no longer shows statistically significant further deterioration — a “floor plateau” is reached. This plateau effect has been consistently reported in men on GnRH agonists, antagonists, and combined novel endocrine agents (36–38).

Importantly, objective somatic function impairment and subjective sexual distress may dissociate over time. While objective function (libido, erectile function) reaches floor by 12 months and plateaus, patients’ subjective sexual distress, self-esteem impact, and partner relationship burden often peak in the first 18 months, then gradually improve as psychological adaptation develops. This dissociation means that cross-sectional correlations between ADT duration and sexual QoL measures can be confounded by the time-varying psychological adaptation process.

These findings suggest that sexual health counseling and intervention should be initiated EARLY — within the first 1–3 months of ADT — when patients are most vulnerable to rapid decline in sexual function. Interventions after the 12-month plateau may have diminishing returns for objective function, but may still benefit subjective distress and psychological adaptation. The cross-sectional design of our study prevented us from characterizing the longitudinal trajectory of sexual function on ADT. Longitudinal studies with repeated measurements are needed to definitively confirm the phased trajectory and plateau effect in Chinese patients and to examine symptom cluster trajectories as a function of ADT duration.

### 4.3 Symptom clusters and quality of life

Our finding that all five symptom clusters were significantly negatively correlated with functional QoL domains (*r* = −0.630 to −0.167, all *P* < 0.01) confirms the pervasive multi-dimensional impact of symptom burden on patient well-being. Notably, the pain-digestive-nutritional cluster demonstrated the broadest cross-domain impact, correlating significantly with 14 of 15 QoL domains — a finding consistent with the literature on co-occurring gastrointestinal, nutritional, and pain symptoms in cancer populations (34, 39). Recent machine learning approaches have also demonstrated the feasibility of predicting symptom cluster trajectories in prostate cancer patients receiving radiotherapy (40), highlighting the growing methodological sophistication in symptom cluster research. However, this broad association is driven by the combined contributions of three symptom subgroups within this cluster — digestive symptoms, nutritional symptoms, and pain — rather than pain alone. Treating pain as the sole driver would therefore oversimplify a multi-factorial symptom experience: gastrointestinal symptoms impair nutritional intake and physical comfort; nutritional decline is associated with functional deterioration and cancer cachexia (41, 42); and pain directly restricts physical activity and emotional well-being (43).

The finding that cognitive function had the lowest score among functional domains (median 50.00) is notable and warrants clinical attention. This may be related to the direct effects of ADT on the central nervous system, as testosterone plays a neuroprotective role (44, 45). Future studies should explore this relationship using objective neuropsychological assessments. The emotional-energy cluster showed the strongest negative correlation with emotional function (*r* = −0.630, *P* < 0.01), suggesting that psychological interventions targeting anxiety, depression, and fatigue may be particularly beneficial for improving emotional well-being. The sexual function cluster had the narrowest association with QoL domains (6 domains), and its impact was relatively modest in magnitude — a finding consistent with the pattern (46), potentially reflecting both the universal nature of this side effect in the ADT population and patients’ progressive adaptation to its inevitability. An additional consideration concerns the correlation between the PCET-SCAS sexual function cluster and the EORTC QLQ-PR25 sexual interest item. As noted in the Results section, the correlation between these two measures was negligible (*r* = 0.026). This low correlation, however, should be interpreted with caution. The PCET-SCAS sexual function cluster assesses symptom burden related to functional impairments—specifically, reduced libido and erectile dysfunction—whereas the EORTC QLQ-PR25 sexual interest item captures the patient’s subjective desire for sexual activity. These are distinct but related facets of sexual health. In clinical practice, it is not uncommon for patients to report preserved sexual interest despite significant functional limitations, or vice versa, particularly in the context of androgen deprivation therapy where psychological adaptation and partner dynamics may play a substantial role (36, 37). Therefore, the low correlation observed in our study likely reflects the multidimensional structure of sexual health rather than a failure of the PCET-SCAS to measure the intended construct. This interpretation is further supported by the pattern of correlations between the sexual function cluster and other QoL domains: the cluster showed significant associations with overall health status, cognitive function, and treatment-related symptoms (**Table 4** and **5**), indicating that it captures clinically relevant aspects of the symptom experience in this population. Future qualitative studies may help clarify how Chinese patients conceptualize and differentiate between sexual interest and sexual function in the context of ADT, and whether cultural factors differentially influence reporting of these two dimensions.

### 4.4 Independent predictors of quality of life

Multivariate regression analysis identified independent predictors of overall health-related QoL (Global Health Status) and hormonal treatment-related symptoms (QLQ-PR25), as detailed in **Table 6**. The 16 models explained 13.9% to 54.1% of the variance, with symptom clusters emerging as dominant predictors — underscoring the importance of integrated symptom management in ADT care.

Performance status (KPS) was a significant consistent cross-domain predictor in regression models, reinforcing its role as a foundational clinical indicator of QoL in patients undergoing ADT (47, 48).

KPS was a significant predictor of physical functioning, role functioning, and social functioning, but not of emotional, cognitive, or sexual outcomes. This pattern is consistent with KPS being a measure of physical performance status rather than a global indicator of well-being. Clinicians should interpret KPS as a specific indicator of physical capacity rather than a comprehensive proxy for overall QoL.

The urological-sleep cluster was significantly associated with role functioning (β = -0.18, P < 0.05), but not with physical or emotional functioning, suggesting that its impact is primarily on functional capacity rather than general physical or emotional well-being. The non-significant association with physical functioning may reflect the counterbalancing effects of urinary symptoms (which impair physical comfort) and sleep disturbances (which impair energy levels), yielding a net effect that does not reach statistical significance in the multivariate model.

The pain-digestive-nutritional cluster exhibited the largest effect sizes across symptom domains, most notably for appetite loss (*β* = 0.714, *P* < 0.001), pain(*β* = 0.632, *P* < 0.001), and constipation (*β* = 0.482, *P* < 0.001), The co-occurrence of pain, gastrointestinal symptoms, and nutritional decline within a single cluster suggests a common pathophysiological mechanism—likely involving ADT-induced metabolic alterations and reduced physical activity leading to progressive nutritional compromise (39, 41). This cluster also independently predicted physical, role, and social function (all *P* < 0.001), indicating that clinicians should treat it as an integrated target requiring coordinated nutritional, pain, and gastrointestinal assessment rather than isolated management.

The endocrine therapy-related cluster was the primary predictor of hormonal treatment symptoms (*β* = 0.465, *P* < 0.001), with all five clusters collectively contributing to this domain (all *P* < 0.05), suggesting that treatment-related side effect burden reflects cumulative symptom load rather than any single symptom group (49, 50). This cluster also independently predicted emotional function (*β* = −0.125, *P* = 0.038),suggesting that hot flashes and related symptoms may contribute to emotional distress through body image and sleep disruption (49, 51).

Cohabitation status was significantly associated with cognitive function (β = 0.151, P = 0.016). A *post-hoc* exploratory analysis further suggested that this association may be modified by educational attainment.

These findings support a tiered intervention model: universal functional and psychological monitoring for all ADT patients; targeted nutritional, pain, and gastrointestinal management for patients scoring high on the pain-digestive-nutritional cluster; proactive urinary and hormonal symptom counseling from treatment initiation; and lifestyle-based physical activity and family engagement programs. Future studies should systematically document specific ADT agents to enable subgroup analyses and better delineate agent-specific symptom burdens.

We did not apply formal multiple comparison corrections (e.g., Bonferroni, false discovery rate) in this study. This decision was based on the exploratory nature of the research, the transparent reporting of all performed tests regardless of statistical significance, and current methodological recommendations that formal adjustment is unnecessary when all results are reported and interpreted equally without selective emphasis on significant findings. Nevertheless, we acknowledge that the risk of Type I error inflation exists, and our findings should be interpreted as hypothesis-generating rather than confirmatory. Replication in independent samples is needed before drawing definitive conclusions. We note that the consistency of findings across multiple QoL domains (e.g., the pain-digestive-nutritional cluster predicting physical, role, and social functioning) provides some protection against Type I error, as the same pattern would be unlikely to arise by chance across multiple independent outcomes.

### 4.5 Limitations

This study has four principal limitations. First, the cross-sectional design precludes causal inferences, and the PCET-SCAS was developed and initially applied in the same cohort, which may introduce optimization bias. Although the companion validation study reports both EFA and CFA results (21), independent external validation across diverse clinical settings and geographic regions is needed to establish the broader generalizability of the five-cluster model. Longitudinal studies with repeated measurements in independent cohorts are warranted to track the dynamic trajectory of symptom clusters across the disease course (14). Second, we conducted multiple regression analyses across QoL domains without formal correction for multiple testing. While all models demonstrated significant overall fit (F-test, P < 0.001) and the identified associations are clinically plausible, domain-specific findings should be interpreted cautiously and in the context of the overall pattern of results. This approach is consistent with methodological guidance emphasizing transparent reporting and cautious interpretation in exploratory analyses (28, 29). Third, single-center enrollment from a single tertiary-level urban hospital may have introduced selection bias. The study sample was predominantly urban (88.3%) and likely skewed toward patients with more advanced disease and better healthcare access. Consequently, the findings may not be directly applicable to patients managed in community-based, rural, or lower-resource clinical settings, where symptom profiles and access to supportive care may differ substantially. Caution should be exercised when generalizing these findings beyond the study population, and future multicenter studies are needed to confirm the external validity of these results. Fourth, despite the use of consecutive enrollment, eligible patients who declined to participate may have differed systematically from those who enrolled—for example, patients with more severe symptom burden or poorer functional status may have been less willing or able to complete the questionnaire—potentially leading to an underestimation of symptom burden and overestimation of QoL in the broader ADT population.

## 5 Conclusion

In conclusion, this study identified distinct symptom cluster profiles in Chinese patients with prostate cancer undergoing ADT and demonstrated their differential impact on QoL. The pain-digestive-nutritional and emotional-energy clusters emerged as the most consistent predictors across multiple QoL domains. KPS predicted physical, role, and social functioning but not emotional or cognitive outcomes (sexual outcomes were not analyzed in regression due to insufficient data), underscoring its role as a physical performance measure rather than a global QoL indicator. These findings support the development of cluster-targeted supportive care interventions to optimize QoL in this population.

## Data Availability

The raw data supporting the conclusions of this article will be made available by the authors, without undue reservation.
