# Supplementary material for: Symptom cluster profiles and their impact on quality of life in Chinese patients with prostate cancer undergoing ADT: a cross-sectional study
Source: Front Oncol. 2026 Jul 24;16:1867688. doi: 10.3389/fonc.2026.1867688 (PMC13447205; doi:10.3389/fonc.2026.1867688)
Supplement: Supplementary file 1 [file Table1.docx]

**STROBE Statement — Checklist of items that should be included in reports of cross-sectional studies**

Manuscript Title: Symptom Cluster Profiles and Their Impact on Quality of Life in Chinese Patients with Prostate Cancer Undergoing ADT: A Cross-Sectional Study

Journal: Translational Andrology and Urology

Study Design: Cross-Sectional Observational Study

Date: April 24, 2026

Note: This checklist has been completed in accordance with the STROBE statement (von Elm E, et al. PLoS Med 2007;4(10):e296). Each item number refers to the STROBE checklist item for cross-sectional studies. Page/section numbers refer to the submitted manuscript.

| **Item No.** | **Section/Topic** | **Recommendation** | **Location in Paper (Section/Page)** |
| --- | --- | --- | --- |
| 1 | Title and abstract | (a) Indicate the study's design with a commonly used term in the title or the abstract | Title: 'A Cross-Sectional Study'; Abstract Methods: 'This cross-sectional study' |
| 1b | Title and abstract | (b) Provide in the abstract an informative and balanced summary of what was done and what was found | Abstract (p.1): Background, Methods, Results, Conclusion sections |
| 2 | Background/rationale | Explain the scientific background and rationale for the investigation being reported | Introduction (p.2-3), paragraphs 1-3 |
| 3 | Objectives | State specific objectives, including any prespecified hypotheses | Introduction last paragraph: 'Accordingly, this study aimed to: (i)... (ii)... (iii)...' |
| 4 | Study design | Present key elements of study design early in the paper | Methods 2.1: 'This was a cross-sectional descriptive study' |
| 5 | Setting | Describe the setting, locations, and relevant dates, including periods of recruitment, exposure, follow-up, and data collection | Methods 2.1: 'outpatient clinics of Guangdong Provincial Hospital of Chinese Medicine, Guangzhou, China, between January 2025 and December 2025' |
| 6 | Participants | Give the eligibility criteria, and the sources and methods of selection of participants | Methods 2.2: Inclusion/exclusion criteria; Methods 2.6: consecutive enrollment |
| 7 | Variables | Clearly define all outcomes, exposures, predictors, potential confounders, and effect modifiers. Give diagnostic criteria, if applicable | Methods 2.5: all measurement instruments defined; Methods 2.7: dependent/independent variables |
| 8 | Data sources/ measurement | For each variable of interest, give sources of data and details of methods of assessment (measurement). Describe comparability of assessment methods if there is more than one group | Methods 2.5.1-2.5.3: detailed description of all instruments |
| 9 | Bias | Describe any efforts to address potential sources of bias | Methods 2.1: consecutive enrollment; Discussion 4.4: limitations including selection bias |
| 10 | Study size | Explain how the study size was arrived at | Methods 2.3: G*Power 3.1.9.7 sample size calculation (f²=0.15, α=0.05, 1-β=0.80, n=168; obtained n=188) |
| 11 | Quantitative variables | Explain how quantitative variables were handled in the analyses. If applicable, describe which groupings were chosen and why | Methods 2.7: continuous variables expressed as median [IQR]; Kolmogorov-Smirnov normality test |
| 12a | Statistical methods | (a) Describe all statistical methods, including those used to control for confounding | Methods 2.7: Spearman correlation, Kruskal-Wallis H, Mann-Whitney U, multiple linear regression (stepwise) |
| 12b | Statistical methods | (b) Describe any methods used to examine subgroups and interactions | Methods 2.7: separate univariate and multivariate models; VIF for multicollinearity |
| 12c | Statistical methods | (c) Explain how missing data were addressed | Methods 2.7: 'Missing data were handled using complete case analysis; sensitivity analyses... confirmed no significant systematic differences' |
| 12d | Statistical methods (cross-sectional) | (d) If applicable, describe analytical methods taking account of sampling strategy | Methods 2.1-2.2: consecutive convenience sampling described; limitations addressed in Discussion 4.4 |
| 12e | Statistical methods | (e) Describe any sensitivity analyses | Methods 2.7: 'sensitivity analyses comparing participants with and without missing PSA values confirmed no significant systematic differences' |
| 13a | Participants | (a) Report numbers of individuals at each stage of study—e.g. numbers potentially eligible, examined, confirmed eligible, included in the study, completing follow-up, and analysed | Methods 2.3: 190 distributed, 188 valid (98.94% response rate) |
| 13b | Participants | (b) Give reasons for non-participation at each stage | Methods 2.3: '190 questionnaires were distributed, and 188 valid questionnaires were collected' |
| 13c | Participants | (c) Consider use of a flow diagram | Not included (consecutive enrollment with very high response rate 98.94%; no flow diagram required) |
| 14a | Descriptive data | (a) Give characteristics of study participants (e.g. demographic, clinical, social) and information on exposures and potential confounders | Results 3.1, Table 1: full demographic and clinical characteristics |
| 14b | Descriptive data | (b) Indicate number of participants with missing data for each variable of interest | Table 1: PSA missing data reported (initial PSA: n=35 missing; current PSA: n=28 missing) |
| 15 | Outcome data | Report numbers of outcome events or summary measures | Results 3.2 (Table 2): symptom cluster scores; Results 3.3 (Table 3): QoL scores |
| 16a | Main results | (a) Give unadjusted estimates and, if applicable, confounder-adjusted estimates and their precision (e.g., 95% confidence interval). Make clear which confounders were adjusted for and why they were included | Results 3.5: univariate analysis; Results 3.6, Table 6: multivariate regression with β, 95%CI, P-value |
| 16b | Main results | (b) Report category boundaries when continuous variables were categorized | Table 1: age, BMI, PSA, KPS all categorized with stated boundaries |
| 16c | Main results | (c) If relevant, consider translating estimates of relative risk into absolute risk for a meaningful time period | Not applicable (cross-sectional regression study, not cohort/RCT) |
| 17 | Other analyses | Report other analyses done—e.g. analyses of subgroups and interactions, and sensitivity analyses | Methods 2.7 & Results 3.5: sensitivity analysis for missing PSA; separate domain-specific regression models |
| 18 | Key results | Summarise key results with reference to study objectives | Discussion introduction (p.9): 'we identified five distinct symptom clusters...' |
| 19 | Limitations | Discuss limitations of the study, taking into account sources of potential bias or imprecision. Discuss both direction and magnitude of any potential bias | Discussion 4.4: five principal limitations (cross-sectional design, single-instrument, single-center, convenience sampling, urban tertiary bias) |
| 20 | Interpretation | Give a cautious overall interpretation of results considering objectives, limitations, multiplicity of analyses, and other relevant studies | Discussion 4.1-4.3: systematic contextualization within literature; Conclusion |
| 21 | Generalisability | Discuss the generalisability (external validity) of the study results | Discussion 4.4 Limitation 3: 'single tertiary-level hospital... caution should be exercised when generalizing' |
| 22 | Funding | Give the source of funding and the role of the funders for the present study and, if applicable, for the original study on which the present article is based | Funding section (p.15): five funding sources listed |

* Items 13c and 16c are not applicable to this study design. All other 22 items have been addressed.

For the complete STROBE guidelines, see: https://www.strobe-statement.org/
