# Supplementary material for: Symptom cluster profiles and their impact on quality of life in Chinese patients with prostate cancer undergoing ADT: a cross-sectional study
Source: Front Oncol. 2026 Jul 24;16:1867688. doi: 10.3389/fonc.2026.1867688 (PMC13447205; doi:10.3389/fonc.2026.1867688)
Supplement: Supplementary file 2 [file Table2.docx]

An initial 50-item pool was generated from systematic literature review and expert consultation, refined via a three-round Delphi study involving 30 experts (mean clinical experience: 26.8 ± 10.2 years; 93.3% with associate senior or higher titles) based on predefined content validity criteria (mean importance > 4.0, full-score ratio > 30%, coefficient of variation < 0.20), yielding 26 items. After pilot testing in 30 patients for readability and face validity, a formal validation study in 188 patients confirmed the final 18-item, five‑cluster structure. The psychometric properties, including exploratory and confirmatory factor analyses and reliability estimates, are reported in the companion validation paper^[21]^.

**Supplementary Table S1. Item composition, descriptive statistics, and factor loadings of the PCET-SCAS (N=188)**

| **Symptom Cluster** | **Item No.** | **Item Description** | **Factor Loading** | **Cumulative Variance Contribution Rate（%）** | **Cronbach's** |
| --- | --- | --- | --- | --- | --- |
| 1. Urological-Sleep | 1 | Frequent urination | 0.834 | 19.355 | 0.812 |
|  | 2 | Nocturia / Increased nighttime urination | 0.797 |  |  |
|  | 3 | Urinary urgency | 0.769 |  |  |
|  | 4 | Urinary leakage / Urinary incontinence | 0.635 |  |  |
|  | 5 | Incomplete bladder emptying | 0.629 |  |  |
|  | 6 | Difficulty falling asleep or frequent nighttime awakening | 0.565 |  |  |
| 2. Pain-Digestive-Nutritional | 7 | Poor appetite / Loss of appetite | 0.876 | 33.111 | 0.679 |
|  | 8 | Weight loss | 0.736 |  |  |
|  | 9 | Generalized body pain | 0.672 |  |  |
|  | 10 | Constipation | 0.437 |  |  |
| 3. Emotional-Energy | 11 | Feeling nervous | 0.880 | 46.373 | 0.813 |
|  | 12 | Feeling sad | 0.864 |  |  |
|  | 13 | Lack of energy | 0.542 |  |  |
|  | 14 | General fatigue / Tiredness | 0.532 |  |  |
| 4.Endocrine Therapy-Related | 15 | Easy sweating / Profuse perspiration | 0.898 | 57.037 | 0.858 |
|  | 16 | Hot flashes | 0.896 |  |  |
| 5. Sexual Function | 17 | Decreased frequency of morning erections | 0.898 | 66.102 | 0.859 |
|  | 18 | Reduced libido and sexual desire | 0.861 |  |  |

*Notes: Factor loadings are from exploratory factor analysis (EFA) with principal axis factoring and promax rotation. All loadings > 0.60. SD, standard deviation; ICC for test-retest reliability = 0.717 (95% CI: 0.642–0.801). Detailed scoring rules are described in the Methods section.*
